# Incontinence of Urine in Children

**Published:** 1879-10

**Authors:** 


### INCONTINENCE OF URINE IN CHILDREN.

At the Harveian Society of London, Dr. Farquharson read a paper on this subject. He referred to the subject under three headings: 1st, Cases observed in children of pale, weakly organizations, in which there is no doubt some weakened condition of the sphincter vesicae, or of the nervous centres in the lumbar cord. Tonic remedies, especially small doses of wine, will act with good effect. 2d, Cases of greater severity, usually dating from soon after birth, and here it is necessary to make a distinction between the enuresis by day and that by night, for the latter frequently resists all medical treatment, departing often spontaneously about the period of puberty. Belladonna, which acts upon the unstriped muscular tissue, gives the best results in such cases. It is necessary to give full doses. Ergot proved disappointing, and santonian is without influence. Class three includes those cases in which the incontinence is truly a neurosis. Here galvanism is especially indicated, and blistering over the fifth lumbar vertebra, where modern experiment has demonstrated the motor centre to be situated. The recently proposed plan of excluding meat from the dietary was not found of much service.

